# Diaphyseal femoral fracture due to severe vitamin D_3_ deficiency and low parathyroid hormone levels on long-term hemodialysis: a case report

**DOI:** 10.1007/s11657-020-00849-7

**Published:** 2020-11-12

**Authors:** Masaki Hatano, Izuru Kitajima, Kazuya Isawa, Yutaka Hirota, Tatsuya Suwabe, Junichi Hoshino, Naoki Sawa, Masaki Nakamura, Seizo Yamamoto, Yoshihumi Ubara

**Affiliations:** 1 Department of Orthopaedic Surgery, Toranomon Hospital, 1-3-1 Kajigaya, Takatu-ku, Kawasaki-shi, Kanagawa 213-8587 Japan; 2 Department of Nephrology Center, Toranomon Hospital, 1-3-1, Takatsu, Kawasaki, Kanagawa 212-0015 Japan; 3 Okinaka Memorial Institute for Medical Research, Toranomon Hospital, Tokyo, Japan

**Keywords:** Atypical femoral fracture, Diaphyseal femoral fracture, Bone histomorphometry, Long-term hemodialysis, Parathyroid hormone, Surgical parathyroidectomy, Vitamin D_3_ deficiency, Osteomalacia, Osteitis fibrosa

## Abstract

**Introduction:**

Currently, there are no reports of diaphyseal femoral fracture equivalent to atypical femoral fractures (AFFs) in patients receiving long-term hemodialysis (HD).

**Case report:**

A 56-year-old Japanese man receiving long-term HD for 34 years was admitted to our hospital due to a delay in postoperative healing. The patient began maintenance hemodialysis at 22 years of age. The patient then underwent surgical parathyroidectomy (PTX) for secondary hyperparathyroidism at 43 years of age, which resulted in decreased levels of parathyroid hormone (PTH). Thereafter, this patient’s serum 1,25(OH)_2_ D_3_ level was very low because active vitamin D_3_ derivative was not administered. At 54 years of age, a transverse fracture of the femoral shaft equivalent to AFF occurred. Surgery with open reduction and internal fixation using intramedullary nailing was performed; however, the delay of postoperative healing continued for 16 months. A left iliac crest bone biopsy was performed and showed osteoid-like lesion and an increase of woven bone. The patient received active vitamin D_3_ derivative and recombinant human PTH (1–34) derivative. Twenty-nine months after the first surgery, a reoperation was performed. Simultaneously, a right iliac crest bone biopsy was performed. Bone morphometrical improvement was confirmed. Six months after resurgery, the bone union was achieved.

**Summary:**

Severe vitamin D_3_ deficiency and decreased levels of PTH may induce a higher osteoid state and an increase of woven bone, which may then attribute to the development of diaphyseal femoral fracture and impairment of postoperative bone healing. It is hypothesized that treatment with active vitamin D_3_ and teriparatide acetate may be a therapeutic option via the accelerated formation of lamellar bone for refractory diaphyseal femoral fracture of long-term dialysis.

**Supplementary Information:**

The online version contains supplementary material available at 10.1007/s11657-020-00849-7.

## Introduction

Mineral abnormalities such as renal osteodystrophy (ROD) are common in patients with chronic kidney disease (CKD) [1]. Previously, the optimal diagnostic test for specific classification of ROD was bone biopsy with bone histomorphometry [2]. Recent studies, however, have shown that serological bone turnover markers are also a useful tool for predicting bone histopathology in patients suffering CKD [1, 3, 4]. The evaluation of such markers is now considered to be essential as it provides vital information required to determine treatment plans for these patients.

Long-term use of bisphosphonates (BPs) has been reported to be the primary cause of femoral stress fractures, also known as atypical femoral fractures (AFF) [5]. The pathogenesis of AFF, however, remains unclear and has been reported to be developed on non dialysis patients. Currently, there are no reports investigating diaphyseal femur fractures in a radiograph equivalent to AFF in patients suffering CKD receiving long-term hemodialysis (HD). In the present study, a patient on long-term HD for 34 years with a history of surgical parathyroidectomy (PTX) presented with poor postoperative healing after experiencing diaphyseal femur fractures. Furthermore, this patient experienced severe vitamin D_3_ deficiency during long-term HD and decreased levels of parathyroid hormone (PTH). We report that bone histomorphometrical analysis in conjunction with evaluation of serological bone turnover markers was able to clarify the pathogenesis of this patient’s bone disease.

### Case presentation

A-56-year-old Japanese man on long-term HD for 34 years was admitted to our hospital for additional treatment for diaphyseal femoral fracture and delay in postoperative healing.

The patient began maintenance dialysis at the age of 22; however, the primary renal disease was unknown. Surgical PTX without autotransplantation was performed for secondary hyperparathyroidism at the age of 43. Thereafter, the patient’s level of intact parathyroid hormone (PTH) was less than 10 pg/mL. At that time, the patient was receiving hemodialysis three times a week for four hours by using dialysate Ca concentration of 3.0 mEq/L and was being prescribed sevelamer hydrochloride (5.25 g/day) and lanthanum carbonate hydrate (0.75 g/day) to treat hyperphosphatemia, precipitated calcium carbonate (2.5 g/day) to treat hypocalcemia, and menatetrenone (15 mg/day) as adjunctive therapy for osteoporosis. However, vitamin D_3_ derivative had not been prescribed.

**Fig. 1:**
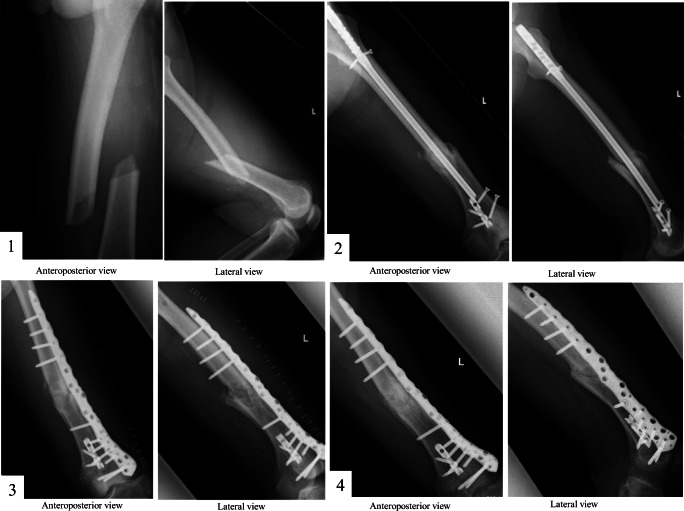
Frontal and lateral radiograph of the left femur. (1), left femoral shaft transverse fracture; (2), bone fixation failure with pseudarthrosis; (3), open reduction and fixation performed using an angular stability plate associated with autogenous bone graft from the patient’s left iliac bone; (4), successful bone union

At the age of 54, the patient felt left thigh pain and experienced walking difficulty without any precipitating cause but did not fall. This pain and walking difficulty were worsening each day. One week later, radiograph showed a transverse fracture of the femoral shaft with spike formation on the postero-medial side, and left diaphyseal femoral fracture was diagnosed (Fig. 1 (1)). The patient underwent open reduction and antegrade surgery for internal fixation, using intramedullary nailing. Three weeks after surgery, partial load was added, and 6 weeks after surgery, full weight load was added. However, this patient still could not walk due to pain, and radiographs showed delayed bone union (Fig. 1 (2)). Sixteen months after this first surgery, postoperative healing was still not achieved. Therefore, the patient was admitted to our hospital for further therapeutic options.

On admission, the patient was 163.9 cm tall and weighed 63.0 kg. Laboratory data (Table 1) was as follows: calcium, 9.0 mg/dL; phosphate, 6.0 mg/dL; alkaline phosphatase (ALP), 193 IU/L (JSCC method, normal range 117 to 350); bone ALP (BAP), 8.8 μg/L (normal range 3.8 to 22.6); tartrate-resistant acid phosphatase-5b (TRAP-5b), 267 mU/dL (normal range 250 to 760); intact PTH, 9 pg/mL (normal range 25 to 117), whole PTH, 12 pg/mL (normal range 9–39); 25-hydroxyvitamin D, 11.5 ng/mL (normal > 20); 1.25-dihydroxyvitamin D_3_, < 4.0 pg/mL (normal range 20–60); osteocalcin, 50.1 ng/mL (normal range 8–33); and undercarboxylated osteocalcin (ucOC), 16.0 ng/mL (normal < 4.5).

**Table 1:** Laboratory data

	First bone biopsy	First postoperative month 3	Second bone biopsy	Second postoperative month 3	Second postoperative month 6	Reference range
BAP	8.8	20.4	14.2	13	18.1	3.7–20.9
TRACP-5b	267	504	411	458	461	170–590
25O-hydroxyvitamin D (nmol/L)	11.5	NM	12.2	10.6	10	> 20
1,25-dihydroxyvitamin D (pg/mL)	< 4	NM	12	30	15	20–60
Osteocalcin (ng/mL)	50.1	61.8	111.1	206.5	181.3	8.4–33.1
ucOC (ng/mL)	15.95	15.22	NM	48.5	49.09	< 4.5
Intact PTH (pg/mL)	9	7	4	4	4	15–65
Whole PTH (pg/mL)	12	< 6	7	< 5.5	5.6	9–39
Total protein (g/dL)	7.5	7.8	7.5	7.3	7.4	6.9–8.4
Albumin (g/dL)	4.2	4	3.9	4.1	4.1	3.9–5.2
Calcium (mg/dL)	9	7.8	8.5	10.9	9.6	8.7–10.1
Phosphate (mg/dL)	6	5.1	5.6	7.4	4.5	2.8–4.6
Alkaline phosphatase (U/L)	138	298	207	64	72	38–113

*BAP* bone alkaline phosphatase, *TRACP-5b* tartrate-resistant acid phosphatase 5b, *ucOC* undercarboxylated osteocalcin, *NM* no measurement

### Clinical course 1

Open reduction and fixation were performed using angular stability plate associated with autogenous bone graft from the patient’s left iliac bone (Fig. 1 (3)). Postsurgical therapy was then conducted for the affected limb, using non-weight bearing methods. Simultaneously, biopsy of the left iliac crest bone was performed to determine the pathogenesis of both diaphyseal femur fractures and impaired bone healing. Bone histomorphometrical analysis was performed by Mrs. Akemi Ito of the Ito Bone Science Institute (Niigata, Japan). Tetracycline double labeling was not performed, because the patient hoped emergent surgery.

### First bone histomorphometric examination

**Fig. 2:**
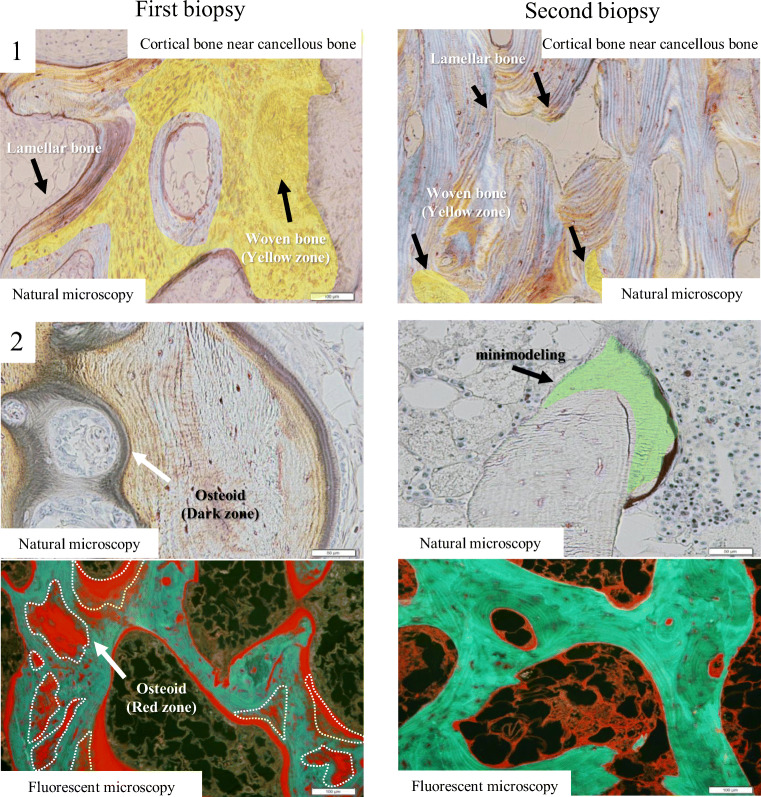
Natural light microscopic analysis. (1), the 1st iliac cortical bone near a cancellous bone section, with woven bone in the cortical bone near cancellous bone (yellow zone). The 2nd iliac cortical bone near cancellous bone with a decrease in woven bone in the cortical bone near cancellous bone and formation of lamellar bone; (2), natural light and fluorescent microscopic analysis of the 1st iliac trabecular bone section. An increase in the amount of osteoid, along with increased thickness of osteoid seam width, and a low calcification area on the bone surface and in the bone, was observed. The 2nd iliac trabecular bone section. The trabeculae bone consisted of minimodeling with minimal to no calcification

The frame of the cortical bone was thin, and the majority of the trabecular bone consisted of cancellous bone. Bone histomorphometric analysis for cancellous bone was measured (Table 2). Trabecular bone volume (BV/TV), trabecular thickness (Tb.Th), and trabecular unit wall thickness (Th.W) were increased by 32.49%, 160.4 μm, and 48.33 μm, respectively, compared with an age-matched reference range according to the report by Reccker RR et al. [6]. Woven bone (Wo.V/BV) of the cancellous bone occupied 2.47% of bone volume, while cortical bone near the cancellous bone occupied 35.3% of bone volume (Fig. 2 (1)). All osteoid markers including osteoid volume to tissue volume (OV/TV), osteoid volume to bone volume (OV/BV), osteoid surface (OS/BS), and osteoid thickness (O.Th) were increased by 4.71%, 14.5%, 66.56%, and 17.45 μm, respectively (Fig. 2 (2)). Fibrous tissue volume to total volume (Fb.V/TV) and eroded surface to bone surface (ES/BS) were increased by 1.08% and 29.98%, respectively. Osteoblasts (Obs) were increased in number by 33.48/mm^2^ and showed cuboidal cytoplasm indicative of an increase in activity. Multinucleated osteoclasts (N.Mu.Ocs) were located in Howship’s lacuna (resorption bay) with deep infoldings, and osteoclast surface to bone surface (Oc.s/BS) was increased by 13.22% with an increase in both number (2.47/mm^2^) and size.

**Table 2:** Histomorphometrical analysis of the 1st and 2nd iliac crest

Parameter		Ratio or abbreviation	Unit	Measured value (1st bone biopsy)	Measured value (2nd bone biopsy)	Normal range
Bone volume	Bone volume	BV/TV	%	32.49	13.71	21.1 ± 3.2
	Trabecular thickness	Tb.Th	μm	160.4	72.42	144.5 ± 17.1
	Wall thickness	W.Th	μm	48.33	29.01	43.2 ± 2.9
Osteoid	Osteoid volume	OV/TV	%	4.71	1.15	0.1~1.0
	Osteoid volume	OV/BV	%	14.5	8.36	4.9 ± 1.2
	Osteoid surface	OS/BS	%	66.56	24.73	23.2 ± 3.4
	Osteoid thickness	O.Th	μm	17.45	12.18	11.6 ± 2.0
	Osteoblast number	N.Ob/BS	N/mm	33.48	2.76	
Resorption	Eroded surface	ES/BS	%	29.98	12.26	5.6 ± 1.7
	Multinucleated osteoclast number	N.Mu.Oc/BS	N.mm	2.47	0.26	
	Osteoclast surface	Oc.s/BS	%	13.22	2.01	
	Fibrous volume	Fb.V/TV	%	1.08	0.04	0
Woven bone	Woven bone (cancellous bone)	Wo.V/BV	%	2.48	1.34	
	Woven bone (cortical bone)	Wo.V/BV	%	35.4	9.33	
	Woven and mineralized bone volume (cancellous bone)	Wo.Md.BV	μm^2^	198,535.01	113,729.7	
	Woven and mineralized bone volume (cortical bone)	Wo.Md.BV	μm^2^	1,456,549.83	645,146.89

### Diagnosis

The patient was diagnosed with osteitis fibrosa due to Fb.V/TV of 1.08% (> 0.5%) and OV/BV of 14.5% (< 15%) according to Sherrard’s classification of ROD [7]. This patient’s osteoid volume (OV/BV) of 14.5% did not reach to limit of osteomalacia (15%) but was increased compared with the normal range value (4.9 ± 1.2) [8]. Osteomalacia-like lesion was definite. Increased activity of both Obs and Ocs was also noted. Fb.V/TV of 1.08% (> 0.5%) and an increase of woven bone imply osteitis fibrosa, which suggested hyperparathyroidism due to higher serum levels of intact PTH [9]; however, the patient’s serum levels of intact PTH were very low with post-PTX. Although this pathogenesis is complex, 1,25-dihydroxyvitamin D_3_ deficiency–related osteomalacia-like lesion and an increase of woven bone were considered to be responsible for the delay in postoperative healing.

### Clinical course 2

The patient began treatment with active vitamin D_3_ derivative (alfacalcidol) at a dose of 0.5 μg/day, in conjunction with recombinant human PTH (1–34) derivative (teriparatide acetate) at 56.5 μg/week, and continues being used for 18 months until today. Nine months later, bone union was still not achieved. A third operation was then performed to decorticate the pseudarthrosis associated with the autogenous bone graft from the patient’s right iliac bone. Simultaneously, bone rebiopsy of the right iliac crest was performed 29 months after the first surgery.

### Second bone histomorphometric analysis

Compared with the first bone biopsy, BV/TV, Tb.Th, and W.Th were decreased to 13.71%, 72.42 μm, and 29.01 μm, respectively. However, trabecular bone consisted of predominantly lamellar bone, and woven bone of cancellous bone was decreased from 2.47 to 1.3%. Furthermore, woven bone volume of cortical bone near cancellous bone was decreased from 35.3 to 9.3% (Fig. 2 (1)). This may indicate that abnormal woven bone was decreased, and consequently, total bone volume was decreased, while healthy lamellar bone was increased.

Compared with the first biopsy, OV/TV, OV/BV, OS/BS, and O.Th improved to 1.15%, 8.36%, 24.73%, and 12.18 μm, respectively. In addition, bone formation by minimodeling, characterized by the lack of precedent bone erosion by osteoclasts, was visible (Fig. 2 (2)) [10]. Both Fb.V/TV and ES/BS decreased by 0.04% and 12.26%, respectively. The Obs had flattened cytoplasm suggesting low activity, and their numbers had decreased to 2.76/mm^2^. Multinucleated osteoclasts (N.Mu.Oc/BS) and osteoclast surface (Oc.s/BS) were decreased (0.26/mm^2^ and 2.01%, respectively), and osteoclast size was decreased. An improvement in bone histomorphometric was confirmed.

Biochemically, the bone turnover markers, osteocalcin and BAP, increased in response to treatment compared with the first operation. Serum intact PTH and whole PTH remained low. Also, compared with the first surgery, 1,25-dihydroxyvitamin D_3_ was increased.

Six months after the third surgery, the bone fracture was in union (Fig. 1 (4)).

## Discussion

Chronic kidney disease–mineral and bone disorder (CKD–MBD) is a complex disorder of both bone and mineral metabolism. Several studies have shown that the risk factors associated with AFF include glucocorticoid, rheumatoid arthritis, and femoral curvature [11, 12]. Currently, ROD is associated with bone fragility and ROD due to long-term HD is considered to be the primary cause of stress fractures [13, 14]. Therefore, it is necessary to accurately evaluate ROD and diagnose the specific type of ROD. According to histological features of bone biopsies [7], ROD is classified as follows: osteitis fibrosa, osteomalacia, and a mixed, mild, or adynamic disease. Previous studies have reported that circulating levels of intact PTH and bone turnover markers are useful tools for predicting bone histopathology [1, 3, 4]. Both BAP and intact PTH are directly correlated with bone formation parameters, and BAP levels are positively correlated with serum levels of intact PTH in patients undergoing HD [1, 4]. In addition, tartrate-resistant acid phosphatase 5b (TRAP-5b) has been reported to be a marker of bone resorption in patients receiving long-term HD [3].

The patient in the current investigation had low levels of intact PTH (< 10 pg/mL) following PTX. This low level of intact PTH continued for 14 years post-PTX. In this particular case, the presence of adynamic bone would be expected, as adynamic bone is caused by hypoparathyroidism. Hypoparathyroidism is typically characterized by hypocellular bone surfaces and markedly reduced bone remodeling. However, it was surprising to find osteitis fibrosa in the first bone histomorphology analysis of this case, indicated by an OV/BV of 14.5% (< 15.0%) and Fb.V/TV of 1.08% (> 0.5%), as osteitis fibrosa is caused primarily by hyperparathyroidism [15]. In addition, the patient had very low serum levels of 1,25-dihydroxyvitamin D_3_. Consequently, it was speculated that severe vitamin D_3_ deficiency in combination with low serum levels of intact PTH following total PTX may have contributed to the bone abnormalities and pseudarthrosis formation, as characterized by postoperative healing failure in this patient. Such speculations are supported by findings of previous, similar case studies [16, 17]. In an attempt to improve postoperative healing, the patient was administered active vitamin D_3_ derivative, to address vitamin D_3_ deficiency, and recombinant human parathyroid hormone (1–34) derivative, to address low postoperative PTH levels.

A common issue among patients undergoing long-term HD is secondary hyperparathyroidism. While considerable advances in medical therapy for secondary hyperparathyroidism have been made, PTX remains the dominant therapeutic tool to address this problem. When compared with other surgical therapies, surgical PTX is considered the superior choice to prevent recurrence of hyperparathyroidism [18]. However, PTX can induce hypocalcaemia and adynamic bone disease [19, 20], and currently, there is no appropriate pharmacotherapy for low levels of PTH following surgical PTX.

Diaphyseal femoral stress fracture or AFF is predominantly caused by long-term treatment with BPs [21]. BPs are potent inhibitors of osteoclast-mediated bone resorption. Long-term use of BPs leads to an accumulation of bone microdamage and suppression of bone turnover, both of which may attribute to AFF. It has been shown that the BP, alendronate, can inhibit normal repair of bone microdamage and cause an accumulation of microdamage [22, 23]. Finally, a previous report suggested that the presence of osteoclasts and resorption were increased in bone biopsies of patients currently being treated with BPs [24]. There was not the report of diaphyseal femoral fracture on patients receiving long-term HD thus far, although femoral neck fracture has been reported.

A previous study has reported the beneficial effects of the recombinant human PTH (1–34), teriparatide, in bone healing following surgery due to AFF [25]. Several studies have also shown the beneficial effects of teriparatide in regard to bone healing in patients undergoing long-term, chronic HD [26]. Low-frequency teriparatide administration has been reported to result in the formation of thicker trabeculae via bone remodeling and minimodeling in young adult male mice [27]. Minimodeling has been reported to have a close relation with bone reformation in hypoparathyroid patients [10]. The findings of the present investigation support these earlier reports, as the second bone biopsy of the patient showed that treatment with teriparatide led to the trabeculae consisting of lamellar bone and minimodeling.

Osteitis fibrosa has been reported to being developed on patients with very high PTH levels. Sherrard et al. reported that fibrous volume (Fb.V/TV) of 57 patients with osteitis fibrosa due to very high PTH levels was 2.2 ± 0.3(%) [7]. Compared with their report, Fb.V/TV of our patient showed lower value with 1.08% and was near normal limit of 0.5%. Originally, dialysis patients with low serum PTH levels will result in adynamic bone when vitamin D_3_ is supplemented. However, we believe that the continuation of severe vitamin D_3_ deficiency in combination with low serum levels of intact PTH induces an increase of osteoid due to mineralization loss and may induce an inappropriate activation of osteoclast and osteoblast leading to mild osteitis fibrosa.

Osteocalcin and ucOC are sensitive markers of bone formation, and these two markers’ value of this patient was always high compared with normal range and increased parallel to treatment with active vitamin D3 derivative and recombinant human PTH (1–34) derivative. This indicates that bone formation by osteoblast was progressing surely. Osteomalacia is a disease characterized by an increase of unmineralized osteoid volume, which is developed by osteoblast, but cannot be mineralized primarily due to severe vitamin D3 deficiency. Higher level of osteocalcin and ucOC of this patient at first biopsy imply that osteoid formation by osteoblast was progressing. And after treatment with active vitamin D3 derivative and recombinant human PTH (1–34) derivative, osteoid is mineralized by using active vitamin D3 derivative, and bone formation by osteoblast is accelerated by using recombinant human PTH (1–34) derivative and resulted in lamellar bone formation.

In conclusion, severe vitamin D_3_ deficiency for long-term HD treatment, and a decrease in serum PTH levels following surgical PTX, may induce an increase in osteoid volume and woven bone, ultimately resulting in diaphyseal femur fracture and impaired bone healing. Daily treatment with active vitamin D_3_ and weekly Intermittent PTH stimulation by using teriparatide acetate may be a potential therapeutic option via the accelerated formation of lamellar bone for refractory diaphyseal femur fracture in a patient receiving HD for a long period of time.

## Supplementary information

ESM 1 (PPTX 2438 kb).

## Abbreviations

AFF: Atypical femoral fracture
BAP: Bone alkaline phosphatase
BPs: Bisphosphonates
BV/TV: Trabecular bone volume to total volume
CKD: Chronic kidney disease
CKD–MBD: Chronic kidney disease–mineral and bone disorder
ES/BS: Eroded surface to bone surface
Fb.V/TV: Fibrous tissue volume to total volume
HD: Hemodialysis
N.Mu.Oc/BS: Multinucleated osteoclasts to bone surface
Obs: Osteoblasts
Oc.s/BS: Osteoclast surface to bone surface
OS/BS: Osteoid surface to bone surface
O.Th: Osteoid thickness
OV/BV: Osteoid volume to bone volume
OV/TV: Osteoid volume to tissue volume
PTH: Parathyroid hormone
PTX: Parathyroidectomy
ROD: Renal osteodystrophy
Tb.Th: Trabecular thickness
Th.W: Trabecular unit wall thickness
TRACP-5b: Tartrate-resistant acid phosphatase 5b
ucOC: Undercarboxylated osteocalcin
Wo.Md.BV: Woven and mineralized bone volume
Wo.V/BV: Woven bone volume to bone volume

## References

[1] Sprague SM, Bellorin-Font E, Jorgetti V, Carvalho AB, Malluche HH, Ferreira A, D’Haese PC, Drüeke TB, Du H, Manley T, Rojas E, Moe SM (2016). Diagnostic accuracy of bone turnover markers and bone histology in patients with CKD treated by dialysis. Am J Kidney Dis.

[2] Moe S, Drüeke T, Cunningham J, Goodman W, Martin K, Olgaard K, Ott S, Sprague S, Lameire N, Eknoyan G (2006). Definition, evaluation, and classification of renal osteodystrophy: a position statement from Kidney Disease: Improving Global Outcomes (KDIGO). Kidney Int.

[3] Shidara K, Inaba M, Okuno S, Yamada S, Kumeda Y, Imanishi Y, Yamakawa T, Ishimura E, Nishizawa Y (2008). Serum levels of TRAP5b, a new bone resorption marker unaffected by renal dysfunction, as a useful marker of cortical bone loss in hemodialysis patients. Calcif Tissue Int.

[4] Ureña P, Hruby M, Ferreira A, Ang KS, de Vernejoul MC (1996). Plasma total versus bone alkaline phosphatase as markers of bone turnover in hemodialysis patients. J Am Soc Nephrol.

[5] Shane E, Burr D, Abrahamsen B, Adler RA, Brown TA, Cheung AM, Cosman F, Curtis JR, Dell R, Dempster DW, Ebeling PR, Einhorn TA, Genant HK, Geusens P, Klaushofer K, Lane JM, McKiernan F, McKinney R, Ng A, Nieves J, O’Keefe R, Papapoulos S, Howe TS, van der Meulen MC, Weinstein RS, Whyte MP (2014). Atypical subtrochanteric and diaphyseal femoral fractures: second report of a task force of the American Society for Bone and Mineral Research. J Bone Miner Res.

[6] Recker RR, Kimmel DB, Parfitt MA, Davies KM, Keshawarz N, Hinders S (1988). Static and tetracycline-based bone histomorphometric data from 34 normal postmenopausal females. J Bone Miner Res.

[7] Sherrard DJ, Hercz G, Pei Y, Maloney NA, Greenwood C, Manuel A, Saiphoo C, Fenton SS, Segre GV (1993). The spectrum of bone disease in end-stage renal failure--an evolving disorder. Kidney Int.

[8] Bhan A, Qiu SJ, Rao SD (2018). Bone histomorphometry in the evaluation of osteomalacia. Bone Rep.

[9] Al-Shoha A, Qiu S, Palnitkar S, Rao DS (2009). Osteomalacia with bone marrow fibrosis due to severe vitamin D deficiency after a gastrointestinal bypass operation for severe obesity. Endocr Pract.

[10] Ubara Y, Tagami T, Nakanishi S, Sawa N, Hoshino J, Suwabe T, Katori H, Takemoto F, Hara S, Takaichi K (2005). Significance of minimodeling in dialysis patients with adynamic bone disease. Kidney Int.

[11] Lim SJ, Yeo I, Yoon PW, Yoo JJ, Rhyu KH, Han SB, Lee WS, Song JH, Min BW, Park YS (2018). Incidence, risk factors, and fracture healing of atypical femoral fractures: a multicenter case-control study. Osteoporos Int.

[12] Sasaki S, Miyakoshi N, Hongo M, Kasukawa Y, Shimada Y (2012). Low-energy diaphyseal femoral fractures associated with bisphosphonate use and severe curved femur: a case series. J Bone Miner Metab.

[13] Pimentel A, Ureña-Torres P, Zillikens MC, Bover J, Cohen-Solal M (2017). Fractures in patients with CKD-diagnosis, treatment, and prevention: a review by members of the European Calcified Tissue Society and the European Renal Association of Nephrology Dialysis and Transplantation. Kidney Int.

[14] Hiramatsu R, Ubara Y, Suwabe T, Sumida K, Hayami N, Yamanouchi M, Mise K, Hasegawa E, Hoshino J, Sawa N, Takaichi K (2012). Osteomalacia and insufficiency fracture in a hemodialysis patient with autosomal dominant polycystic kidney disease. Intern Med.

[15] Malluche HH, Monier-Faugere MC (1994). The role of bone biopsy in the management of patients with renal osteodystrophy. J Am Soc Nephrol.

[16] Kanis JA (1981). Osteomalacia and chronic renal failure. J Clin Pathol.

[17] Yajima A, Tsuchiya K, Burr DB, Wallace JM, Damrath JD, Inaba M, Tominaga Y, Satoh S, Nakayama T, Tanizawa T, Ogawa H, Ito A, Nitta K (2019). The importance of biologically active vitamin D for mineralization by osteocytes after parathyroidectomy for renal hyperparathyroidism. JBMR Plus.

[18] Schneider R, Slater EP, Karakas E, Bartsch DK, Schlosser K (2012). Initial parathyroid surgery in 606 patients with renal hyperparathyroidism. World J Surg.

[19] Lorenz K, Ukkat J, Sekulla C, Gimm O, Brauckhoff M, Dralle H (2006). Total parathyroidectomy without autotransplantation for renal hyperparathyroidism: experience with a qPTH-controlled protocol. World J Surg.

[20] Saunders RN, Karoo R, Metcalfe MS, Nicholson ML (2005). Four gland parathyroidectomy without reimplantation in patients with chronic renal failure. Postgrad Med J.

[21] Tamminen IS, Yli-Kyyny T, Isaksson H, Turunen MJ, Tong X, Jurvelin JS, Kröger H (2013). Incidence and bone biopsy findings of atypical femoral fractures. J Bone Miner Metab.

[22] Odvina CV, Zerwekh JE, Rao DS, Maalouf N, Gottschalk FA, Pak CY (2005). Severely suppressed bone turnover: a potential complication of alendronate therapy. J Clin Endocrinol Metab.

[23] Mashiba T, Hirano T, Turner CH, Forwood MR, Johnston CC, Burr DB (2000). Suppressed bone turnover by bisphosphonates increases microdamage accumulation and reduces some biomechanical properties in dog rib. J Bone Miner Res.

[24] Compston J (2011). Pathophysiology of atypical femoral fractures and osteonecrosis of the jaw. Osteoporos Int.

[25] Im GI, Lee SH (2015). Effect of teriparatide on healing of atypical femoral fractures: a systemic review. J Bone Metab.

[26] Sumida K, Ubara Y, Hoshino J, Mise K, Hayami N, Suwabe T, Kawada M, Imafuku A, Hiramatsu R, Hasegawa E, Yamanouchi M, Sawa N, Takaichi K (2016). Once-weekly teriparatide in hemodialysis patients with hypoparathyroidism and low bone mass: a prospective study. Osteoporos Int.

[27] Yamamoto T, Hasegawa T, Sasaki M, Hongo H, Tsuboi K, Shimizu T, Ota M, Haraguchi M, Takahata M, Oda K, Luiz de Freitas PH, Takakura A, Takao-Kawabata R, Isogai Y, Amizuka N (2016). Frequency of teriparatide administration affects the histological pattern of bone formation in young adult male mice. Endocrinology.

