# Supplementary material for: Diaphyseal femoral fracture due to severe vitamin D3 deficiency and low parathyroid hormone levels on long-term hemodialysis: a case report
Source: Arch Osteoporos. 2020 Nov 12;15(1):179. doi: 10.1007/s11657-020-00849-7 (PMC7661412; doi:10.1007/s11657-020-00849-7)
Supplement: Supplementary file 1 — (PPTX 2438 kb). [file 11657_2020_849_MOESM1_ESM.pptx]

## Slide 1
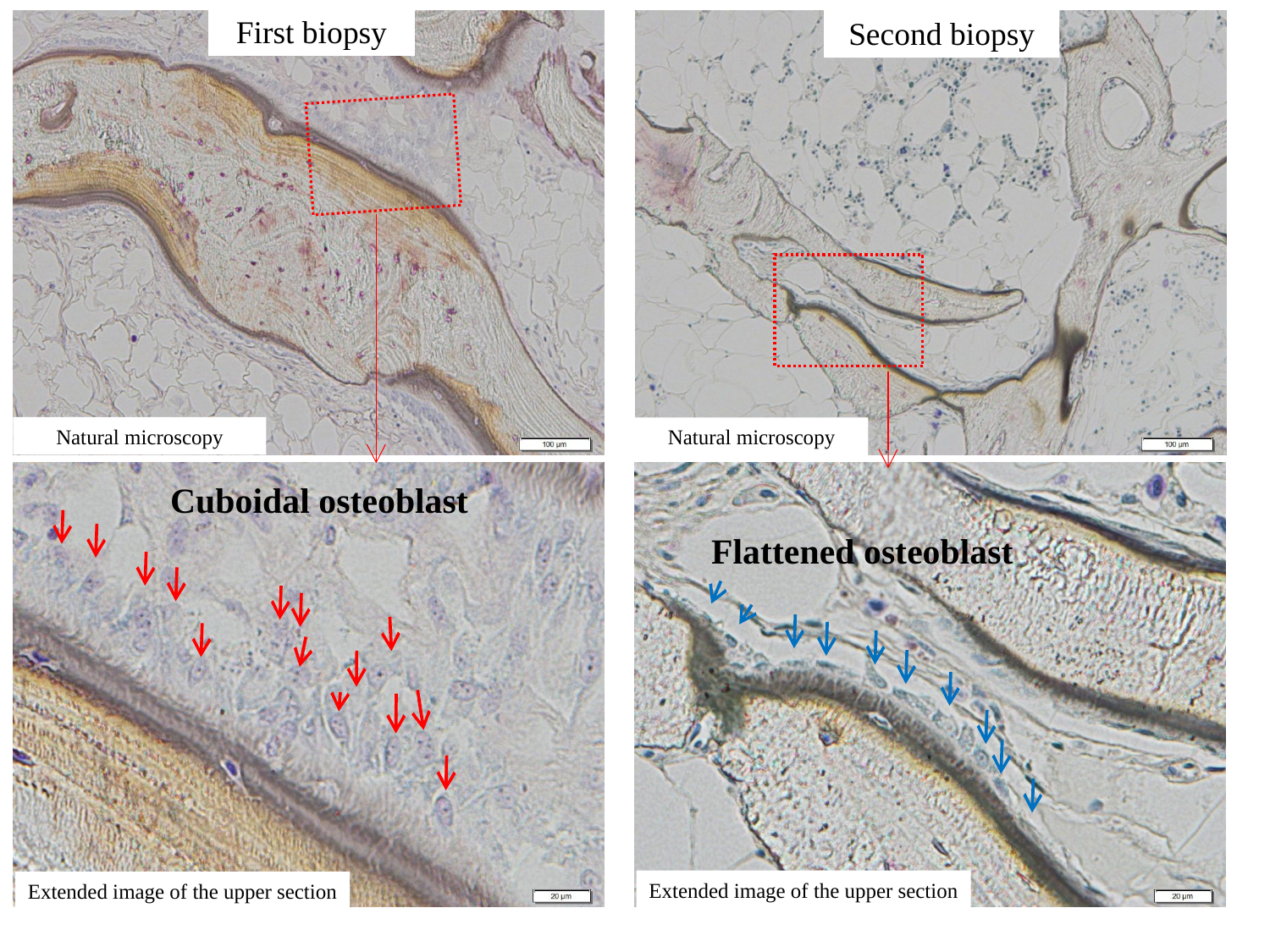

First biopsy
Second biopsy
Natural microscopy
Natural microscopy
Cuboidal osteoblast
Flattened osteoblast
Extended image of the upper section
Extended image of the upper section

## Slide 2
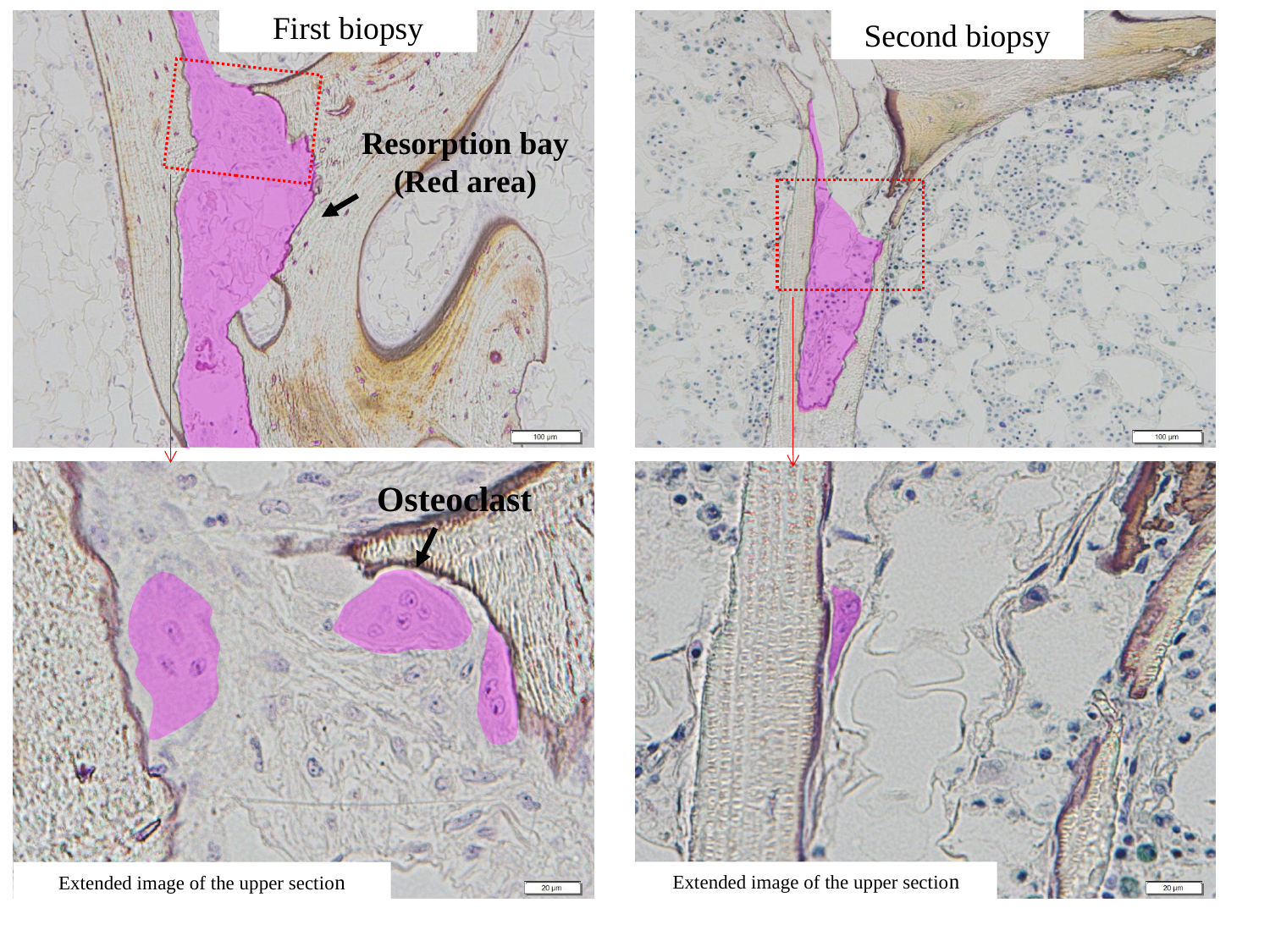

First biopsy
Second biopsy
Resorption bay (Red area)
Osteoclast
Extended image of the upper section
Extended image of the upper section
